# In vivo quantification of [^11^C]BIO-1819578 in non-human primates, a novel radioligand for O-GlcNAcase

**DOI:** 10.1177/0271678X251332487

**Published:** 2025-04-12

**Authors:** Martin Bolin, Sangram Nag, Ryosuke Arakawa, Andrea Varrone, Lars Farde, Laurent Martarello, Maciej A Kaliszczak, Christer Halldin, Anton Forsberg Morén

**Affiliations:** 1 Centre for Psychiatry Research, Department of Clinical Neuroscience, 27106 Karolinska Institutet, & Stockholm Health Care Services, Region Stockholm, Sweden; 2 Sentonix Inc., 1167 Massachusetts Ave., Arlington, MA, USA; 3 BIOGEN MA Inc., Cambridge, MA, USA; 4 Department of Biophysics and Radiation Biology, and HUN-REN TKI, 37637 Semmelweis University, Budapest, Hungary

**Keywords:** Alzheimer, kinetic modelling, O-GlcNAcase, OGA, PET

## Abstract

Neurofibrillary tangles (NFTs), composed of aggregated tau protein, in the brain is a neuropathological hallmark and feature of Alzheimer’s disease (AD) and other tauopathies. One promising approach to prevent tau aggregates is to inhibit O-GlcNAcase (OGA), an enzyme that regulates tau O-GlcNAcylation. [^11^C]BIO-1819578 has emerged as a promising candidate to determine target occupancy of such OGA inhibitor drugs. The aim of this study was to further evaluate the pharmacokinetic properties of [^11^C]BIO-1819578 in non-human primates (NHPs) and to estimate its effective dose. Kinetic compartment analyses of [^11^C]BIO-1819578 binding to OGA in the brain were performed on positron emission tomography (PET) measurements conducted in three cynomolgus NHPs. Whole-body PET measurements were carried out in two NHPs to estimate the effective radiation dose. Both the 1-tissue-compartment (1TCM) and 2-tissue-compartment model (2TCM) could describe the regional time activity curves of [^11^C]BIO-1819578. The 2TCM was the statistically preferred model. The effective radiation dose was estimated to be 0.0033 mSv/MBq. The results showed that [^11^C]BIO-1819578 has suitable characteristics for reliable quantification of OGA using full kinetic modelling. The effective dose was on par with other ^11^C radioligands and is unlikely to pose an issue for human use.

## Introduction

The presence of neurofibrillary tangles (NFTs) in the brain is one of the neuropathological hallmarks of Alzheimer’s disease (AD). These intraneuronal tangles contain fibrillary structures that are formed by the aggregation of hyperphosphorylated microtubular-associated tau protein.^1^ An increase in intracellular tau aggregates is highly linked to more severe disease states of AD and other tauopathies.^2^ These observations have encouraged efforts to develop treatment approaches that can slow down or prevent the formation of pathological tau.^3,4^ Diverse therapeutic strategies have been proposed to reduce tau expression or inhibit the formation of tau aggregates.^5^

In relevant tau transgenic mouse models, inhibition of O-GlcNAcase (OGA) to increase O-GlcNAcylation in the brain has been shown to protect against pathological tau^6–8^ and several selective OGA inhibitor drugs have entered clinical trials.^9–11^ A handful of positron emission tomography (PET) radioligands have been developed for OGA,^12–15^ such tools are not only useful to quantify OGA levels in the living brain but are also instrumental for the direct measurement of OGA level of engagement by drug candidates entering clinical development to establish the relationship between blood concentration and target occupancy.^16^ Understanding the extent to which OGA inhibitors bind to their target is pivotal in selecting a dose and a dose regiment that maximize efficacy and minimize potential side effects, ultimately guiding clinical development decision-making and patient care.^17^

Among these PET radioligands, BIO-1819578 emerged as a promising high affinity OGA inhibitor candidate (Kd = 2.3 nM).^15^ As previously reported BIO-1819578, was radiolabelled with ^11^C either using [^11^C]CH_3_I at the methyl position^15^ and using [^11^C]CO at the amide group^18^ in our laboratory. Considering the higher radiochemical yield and promising pilot results showing favourable brain kinetics and specificity, the [^11^C]CO labelling synthon was selected for further investigations of [^11^C]BIO-1819578^18^ in the NHP reported in this paper.

To date, no kinetic compartment analysis of [^11^C]BIO-1819578 has been performed using a radiometabolite corrected arterial plasma input function. As OGA is expressed in blood cells, full quantification using a metabolite corrected input function is required for valid quantification. The primary objective of the present analysis was to describe and interpret [^11^C]BIO-1819578 kinetics in the NHP brain under baseline condition and post administration Thiamet-G at a dose known to significantly engage OGA.^13^ The secondary objective was to determine the effective radiation dose of [^11^C]BIO-1819578 in NHPs, thereby paving the way for safe human use. The results from this study are intended to provide the foundation for further evaluation in human studies. The future aims are to assess the quantification and test-retest reproducibility in humans to support further use in assessing novel disease modifying therapies directed towards OGA activity.

## Materials & method

The study was approved by the Animal Ethics Committee of the Swedish Animal Welfare Agency (Dnr 10367-2019) and was performed according to “Guidelines for planning, conducting and documenting experimental research” (Dnr 4820/06-600) of Karolinska Institutet. The NHPs were housed in the Astrid Fagraeus Laboratory (AFL), Comparative Medicine, Karolinska Institutet, Solna, Sweden. AFL is accredited by the organisation AAALAC International for its high quality in animal welfare. The research conducted and the reporting which follows is compliant with ARRIVE guidelines.

The present compartment analysis of regional [^11^C]BIO-1819578 binding in NHPs was partly based on data obtained in a previously published PET study.^18^ The following is a short description of the sample and imaging procedure in the present and previous study.

## Radioligand synthesis

[^11^C]BIO-1819578 was synthesized and formulated as reported previously.^18^ The synthesis of [^11^C]-carbonmonoxide ([^11^C]CO) was performed using a modified method from previous literature.^19^ No carrier-added [^11^C]CO2 was produced by bombarding a mixture of nitrogen and 0.5% oxygen gas with 16.5 MeV protons via the ^14^ N(p,α)^11^C nuclear reaction. [^11^C]BIO-1819578 was synthesized by trapping [^11^C]CO at room temperature in a reaction vessel containing the amine precursor (BIO-1952489, 2 mg), methyl palladium(II) chloride complex (8.0 mg), and XantPhos (12 mg) in 400 µL of THF, followed by heating at 110 °C for 400 seconds. After synthesis, 500 µL of DMSO was added, and THF was evaporated using a helium flow. The resulting residue was diluted with 2 mL of sterile water before injected into an HPLC for purification, using a semi-preparative reverse phase ACE column. The desired product [^11^C]BIO-1819578 was eluted with a retention time of 10–11 minutes, diluted with 50 mL sterile water, and passed through a preconditioned SepPak tC18 plus cartridge for isolation. Finally, the product was eluted with ethanol into a sterile vial, sterile filtered, and the radiochemical purity and stability were assessed using analytical HPLC, confirming the identity of the radiolabeled compound through co-injection with an authentic reference standard.

The molar activity of the produced radioligand was 17 ± 6 GBq/μmol (range, 9–29 GBq/µmol) at the time of injection to NHP. The radiochemical purity was >99% at the end of the synthesis. The final product [^11^C]BIO-1819578 formulated in sterile saline was found to be stable with a radiochemical purity of more than 99% for up to 60 min.

## Subjects

For regional brain quantification of [^11^C]BIO-1819578 data obtained from three adult NHPs, two males and one female cynomolgus monkey, were used (average weight 7.5 kg, range: 7.2–8.2 kg).

For the dosimetry study two NHPs, one male (also included in the brain studies) and one female, were used (average weight 5.9 kg, range: 5.3–6.5 kg).

## Drug administration

For the baseline-pretreatment study in one of the three NHPs Thiamet-G was administered intravenously for a duration of 5 minutes, 45 minutes prior to PET start, with a dose of 10 mg/kg and a volume of 1.0 ml/kg. Pharmacokinetic samples, for venous plasma drug concentration, were acquired at -1-, 1-, 15- and 45-minutes relative the Thiamet-G administration.

## Acquisitions

PET imaging was performed on the High Resolution Research Tomograph (HRRT, CTI/Siemens Medical Solutions). List-mode data was reconstructed with 3 D-OSEM with point spread function correction using 10 iterations and 16 subsets, with isotropic voxels of 1.22 mm using a matrix size of 256 ×256 × 207.

### Brain PET measurement

Anaesthesia was induced in the NHPs via intramuscular injection of ketamine (10 mg/kg) and maintained through the administration of a mixture of sevoflurane, oxygen, and medical air with endotracheal intubation. Throughout the PET measurements, the animals’ body temperature, ECG, heart rate, respiratory rate, oxygen saturation, and blood pressure were continuously monitored.

For brain PET experiments the NHP head was immobilized during the entire PET measurement using a specifically designed head fixation device. Prior to PET acquisition, a 6-minute ^137^Cs transmission scan was performed for attenuation and scatter correction purposes. Following an intravenous 10 s bolus-injection of [^11^C]BIO-1819578 (83 ± 5 MBq, 2.0 ± 1.0 µg), list-mode data was then acquired dynamically over a total of 93 minutes and reconstructed into 38 frames (10 s × 9, 15 s × 2, 20 s × 3, 30 s × 4, 1 min × 4, 3 min × 4, 6 min × 12).

A total of six PET scans were conducted, with five at baseline condition and one in a pretreatment setting. Four baseline PET scans were performed in two NHPs and served as a test-retest evaluation of [^11^C]BIO-1819578.

### Dosimetry - Whole body PET measurements

Anaesthesia preparations and ^137^Cs transmission scans were performed in the same manner as for the Brain PET measurements. For each NHP, two PET scans were performed on the same day, each with separate radioligand injections. One scan covered the head to the abdomen (PET scan 1), while the other covered the abdomen to the groin (PET scan 2), to estimate the biodistribution throughout the entire body of the NHP. List-mode data of [^11^C]BIO-1819578 was acquired dynamically over a total of 123 minutes and the 4 D PET data images were reconstructed in 26 frames (1 min × 3, 3 min × 6, 6 min × 17).

The mean injected radioactivity of [^11^C]BIO-1819578 was 80 MBq (PET scan 1: 79 MBq, PET scan 2: 81 MBq) and 81 MBq (PET scan 1: 81 MBq, PET scan 2: 81 MBq) for the two NHPs, respectively. The mean molar activity was 19.6 GBq/μmol (PET scan 1: 14.7 GBq/µmol, PET scan 2:24.5 GBq/μmol) and 13.4 GBq/μmol (PET scan 1: 13.1 GBq/µmol, PET scan 2: 13.6 GBq/μmol), respectively, and the mean injected mass was 1.6 μg (PET scan 1: 1.2 µg, PET scan 2: 2.0 μg) and 2.2 μg (PET scan 1: 2.2 µg, PET scan 2: 2.2 μg), respectively.

### MRI measurements

T1-weighted MR imaging was performed on a 1.5-T GE Signa system (General Electric, Milwaukee, WI, USA) using a 3 D spoiled gradient echo protocol with the following settings: repetition time 21 ms, flip angle 35°; FOV 12.8 cm; matrix 256 × 256 × 128; 1.0 mm slice thickness; 2 number of excitations.

### Arterial blood sampling

Arterial blood was sampled and measured for radioactivity continuously at a speed of 3 ml/min for the first 3 minutes of scanning using an Allogg AB automatic blood sampling machine. Discrete samples were acquired for metabolite and radioactivity measurements at 1, 1.5, 2, 2.5, 3, 5, 15, 30, 60, 75 and 90 minutes post radiotracer administration.

## Analyses

### Radiometabolite analysis

A reverse-phase high-performance liquid chromatography (HPLC) coupled with ultraviolet (254 nm) and radioactivity detection was used for determination of the percentages of radioactivity corresponding to [^11^C]BIO-1819578 and its radioactive metabolites during the course of PET measurement, as reported previously.^18,20^ Arterial plasma samples, obtained at 2, 5, 15, 30, 60, and 75 minutes post-injection, were extracted with acetonitrile to precipitate proteins. Following dilution with water, the extract was injected onto an ACE 5 μm C18 HL column (250 × 10 mm). Analytes were separated by gradient elution at a flow rate of 5.0 mL/min, using acetonitrile (A) and 0.1 M ammonium formate (B) as the mobile phases. The gradient program was as follows: 0 − 4.0 min, (A/B) 40:60 → 90:10 v/v; 4.0 − 6.0 min, (A/B) 90:10 v/v. The fraction of total radioactivity corresponding to the parent radioligand, [^11^C]BIO-1819578, was determined by integrating the area under its chromatographic peak and expressing it as a percentage of the total integrated area of all detected radioactive species.

### Radiometabolite-corrected arterial plasma radioactivity curve

As complete data were not available in all cases due to low signal-to-noise ratio in the radiometabolite analysis, the individual parent fraction estimates were calculated using the Richards function^21^ and a Bayesian population-aided approach. The individual plasma samples and parent fraction estimates were combined to create the radiometabolite-corrected arterial plasma radioactivity curve.

### Protein binding

Using an ultrafiltration method,^20^ the free fraction (f_p_) of [^11^C]BIO-1819578 in plasma was determined for the regional brain quantification study.

### Brain image analysis

The image data were analysed using MatLab 2014 b (Natick, Massachusetts: The MathWorks Inc.) and an in-house imaging analysis pipeline that performed the kinetic and graphical analysis. The volumes of interest were manually drawn on the T1w MR images for each NHP for whole brain and the regional regions: Putamen, Caudate, Frontal Cortex, Occipital Cortex, Hippocampus, Cerebellum, Thalamus. The coregistration of PET data to MR images was performed using SPM12 (http://fil.ion.ucl.ac.uk/spm/). The coregistered volumes were applied to the PET data outside of SPM12 to generate time-activity curves of brain regions for each dynamic PET dataset.

### Kinetic compartment analysis

The analysis included the 1-tissue compartment model (1TCM) and the 2-tissue compartment model (2TCM).^22^ The radiometabolite-corrected arterial plasma radioactivity curve was used as input function. The cerebral blood volume (CBV) was fitted using the 2TCM and then applied across all brain regions. As the primary outcome measure, the total distribution volume (*V*_T_) was calculated. For the 1TCM, *V*_T_ was defined as K_1_/k_2_, while for the 2TCM, it was calculated as K_1_/k_2_ × (1 + k_3_/k_4_). The goodness of fit was determined using the second-order corrected Akaike information criteria (AICc).^23^ Time stability of *V*_T_ was estimated using 2TCM; It was estimated across brain regions during baseline experiments by iteratively calculating *V*_T_ from progressively shortened time series data.

### Graphical analysis

The Ichise Multilinear Analysis MA1^24^ was also used, as it has been proposed as a reliable graphical modelling approach with improved stability in noisy data compared to other graphical approaches such as Logan graphical analysis, to estimate *V*_T_ with t* set to 30 minutes, chosen since it provided good fit for all included regions. The coefficient of determination was calculated between *V*_T_ values from 2TCM and MA1.

### Test-retest variability

The test-retest variability for each region was calculated as: $\mathrm{TRV}=\frac{1}{N}\sum_{i=1}^{N}\frac{\left|{\mathrm{test}}_{i}-{\mathrm{retest}}_{i}\right|}{\left({\mathrm{test}}_{i}+{\mathrm{retest}}_{i}\right)/2}$ , where *N* denotes the number of subjects, and *test_i_* and *retest_i_* are the result values of the quantification method.^25^

### Parametric imaging

Wavelet-aided parametric imaging (WAPI)^26^ was used to generate parametric images of [^11^C]BIO-1819578 *V*_T_ estimates. WAPI employs wavelet filtering to reduce noise and improve the signal-to-noise ratio before applying the Logan’s graphical approach.

### Occupancy estimation after pretreatment with Thiamet-G

Using the revisited Lassen plot,^27^ occupancy was estimated after pretreatment with Thiamet-G.

### Image analysis of the Whole-Body PET

The whole-body PET image data were analysed using PMOD, version 4.2 (PMOD Technologies LLC). Volumes of interest were drawn on high-uptake organs such as the brain, lung, heart, salivary gland, thyroid, kidney, spleen, liver, gallbladder (only in one NHP), urinary bladder, small intestine, bone (lumbar vertebrae) with the help of the CT images for anatomic landmarks. The absorbed radiation dose in humans was predicted with OLINDA/EXM 1.1 software, using the sex average adult reference model.^28^

## Results

### Brain PET data

**Figure 1.**
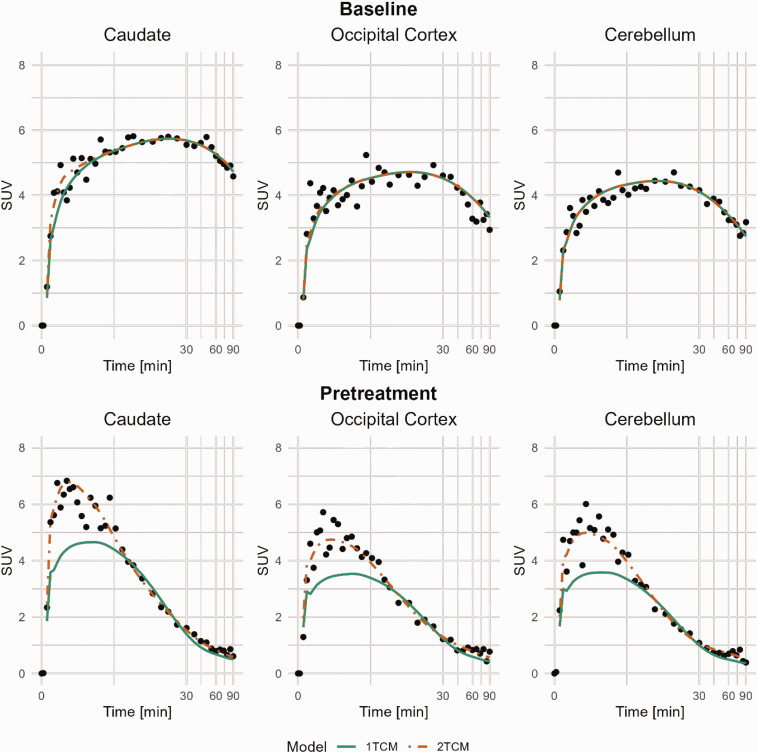
Compartmental-model curve fitting of [^11^C]BIO-1819578 for Caudate, Occipital Cortex and Cerebellum. Data are from baseline and pretreatment PET of NHP3. A logarithmic time scale was chosen to visualise the early peak difference between the models. 1TCM: 1-tissue compartment model; 2TCM: 2-tissue compartment model.

**Figure 2.**
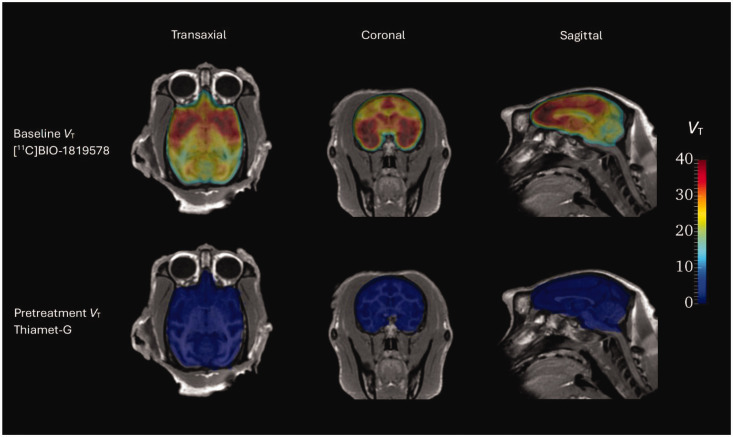
Parametric V_T_ images of [^11^C]BIO-1819578 at baseline and under pretreatment with Thiamet-G. Both sets of data are overlayed on corresponding MR image in NHP3.

All together five baseline PET-measurements were performed in the three NHPs. The time-activity curves for the whole brain peaked at approximately 4.4 SUV on average around 15 minutes post radioligand injection and then decreased to around 3.3 SUV at 90 minutes under baseline conditions. Average regional uptake curves at baseline (n = 5) are presented in Supplemental Figure 1; Regional brain uptake generally peaked at 5–15 minutes post-injection. Figure 1 shows regional time-activity curves in the baseline and pretreatment conditions for NHP 3. Parametric *V*_T_ images from NHP 3 are shown in Figure 2, demonstrating that pretreatment with Thiamet-G reduced the distribution volume in all brain regions.

### Metabolite corrected arterial input function

The time curve for total radioactivity in arterial plasma was generated using the continuous whole blood and discrete blood and plasma samples. Blood to plasma ratio was best estimated using an exponential fitting method. Extrapolation of the parent fraction estimation, using a Bayesian population-aided approach, was carried out, as low signal to noise was observed in the chromatogram analysis after 60–75 minutes post injection. On average, the model estimated that 27% of [^11^C]BIO-1819578 remained unmetabolized in NHP plasma at 60 minutes post-injection (Supplemental Figure 2). The time curve for parent fraction and the blood to plasma ratio was used to correct the total radioactivity in arterial blood to obtain the metabolite corrected arterial input function used in the compartment analysis.

The free fraction was determined to 26%, on average (range 22–30%), for the baseline experiments and the single estimate after pretreatment with Thiamet-G was also 26%. The metabolite corrected arterial input function was not corrected for f_p_.

### Kinetic analysis

At baseline, both the 1-tissue compartment model (1TCM) and the 2-tissue compartment model (2TCM) were able to describe the time-activity curves in most brain regions (Figure 1), and the Akaike information criterion (AICc) could not separate out a preferable model across regions and subjects (Supplemental Table 1).

**Figure 3.**
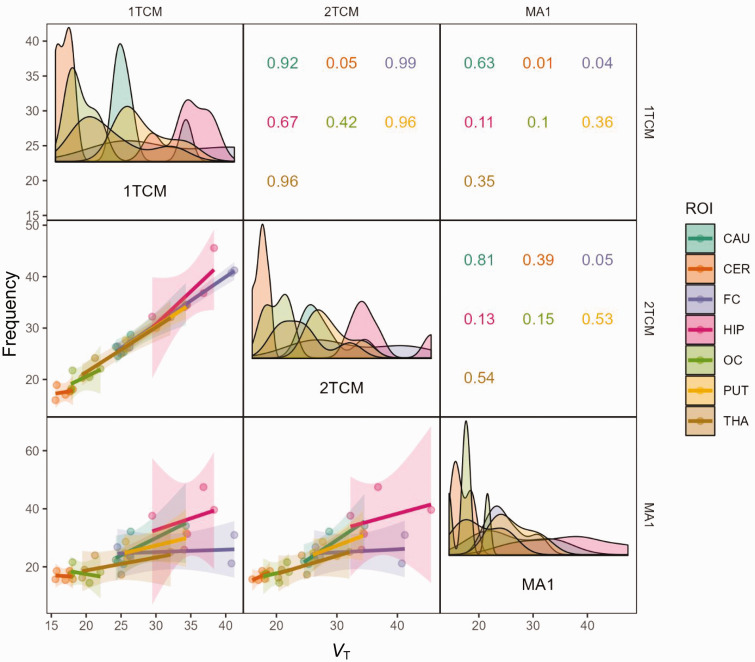
Correlation plot of V_T_ values of the compartmental and graphical models. The diagonal panels show the density plots of individual V_T_ estimates. Panels in the lower left show linear regression data between models. 1TCM: 1-tissue compartment model; 2TCM: 2-tissue compartment model; MA1: Ichise Multilinear Analysis 1; Panels in the upper right show R^2^ values between models for the different regions. CAU: caudate; CER: cerebellum; FC: frontal cortex; HIP: hippocampus; OC: occipital cortex; PUT: putamen; THA: thalamus.

After pretreatment, the 2TCM was the single method that could describe the time-activity curves. Figure 1 presents the model estimates for the Caudate, Occipital Cortex, and Cerebellum in NHP3 at baseline and after pretreatment. Combining the baseline data from the three NHPs, 18 out of 35 regions were better estimated using the 2TCM compared to the 1TCM, based on the AICc score. The diagonal panels in Figure 3 shows the distribution of individual *V*_T_ estimates using different modelling methods. The distributions are the narrowest for Cerebellum and Occipital Cortex and the widest for Frontal Cortex and Hippocampus, indicating the level of accuracy for the different models.

In the analysis of baseline scans, the 2TCM demonstrated that *V*_T_ values across different brain regions converged to within 10% of their final values after 66 minutes of image acquisition (Supplemental Figure 3).

The *V*_T_ values correlated well between the two models, with an overall R^2^ of 0.96 and individual region R^2^ ranging from 0.05 to 0. 99 (Figure 3). For the pretreatment data, all regions were preferably modelled using the 2TCM according to the AICc score, and the R^2^ between the two compartmental approaches was 0.93. After pretreatment with Thiamet-G, an occupancy of 97.6% (calculated using the 2TCM) was observed, with an estimated *V*_ND_ of 2.9 mL·cm^−3^ (Supplemental Figure 4). The individual *V*_T_ values estimated from the 2TCM are presented in Table 1.

**Table 1.** Regional V_T_ values for different models. Presented are median values and the interquartile range.

	Baseline *V*_T_ Values [mL·cm^−3^] median (1^st^ Qu.–3^rd^ Qu.)		
Region	1TCM	2TCM	MA1
Caudate	25.4 (24.5–26.4)	26.4 (25.3–28.7)	22.4 (21.9–32.2)
Cerebellum	17.0 (15.8–17.6)	17.6 (17.0–18.1)	15.8 (15.7–18.6)
Frontal Cortex	26.4 (26.2–40.8)	27.2 (26.5–40.7)	23.9 (23.3–26.8)
Hippocampus	34.5 (34.1–36.8)	34.5 (34.1–36.8)	37.6 (31.3–39.6)
Occipital Cortex	18.0 (17.9–20.5)	20.5 (17.2–21.8)	17.7 (17.2–18.2)
Putamen	25.9 (25.7–30.1)	27.6 (26.1–30.0)	24.8 (23.7–28.6)
Thalamus	21.3 (19.8–25.0)	24.2 (20.9–25.0)	19.0 (17.3–24.0)

### Graphical analysis

**Figure 4.**
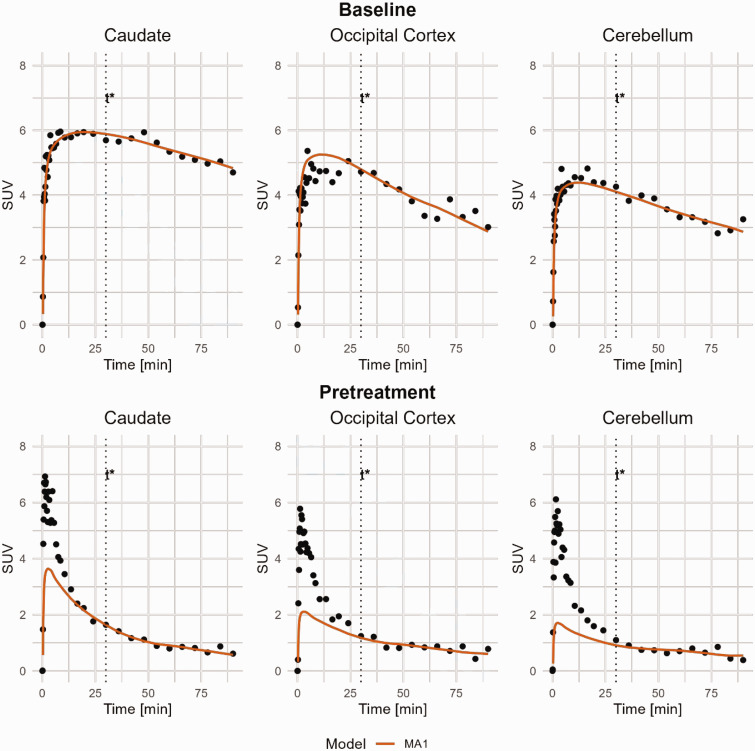
MA1 curve fitting of [^11^C]BIO-1819578 for Caudate, Occipital Cortex and Cerebellum. Data are from baseline and pretreatment PET of NHP3. t* was set to 30 minutes. MA1: Ichise Multilinear Analysis 1.

MA1 estimates examples are shown in Figure 4 and individual *V*_T_ from MA1 are shown in Table 1. The correlation between MA1 and the compartmental models are shown in Figure 3. At baseline, the overall R^2^ was determined to 0.64 between MA1 and 2TCM, ranging from 0.05 to 0.81 for individual regions. MA1 estimated *V*_T_ values were negatively biased by approximately 7% compared to those estimated with 2TCM.

### Test-retest variability

The regional test-retest variability (*N* = 2) results are presented in Table 2. Analysis of the data indicate that the 1TCM demonstrated the lowest overall test-retest variability among the models examined.

**Table 2.** Test-retest variability for regional brain data for different models.

	Test-retest variability [%]		
Region	1TCM	2TCM	MA1
Caudate	5.9	15.0	20.1
Cerebellum	3.6	11.6	9.4
Frontal Cortex	25.5	21.9	12.8
Hippocampus	13.7	15.7	12.1
Occipital Cortex	7.4	14.9	18.4
Putamen	7.8	12.4	9.0
Thalamus	16.2	16.4	14.6

### Whole-Body PET and dosimetry

**Figure 5.**
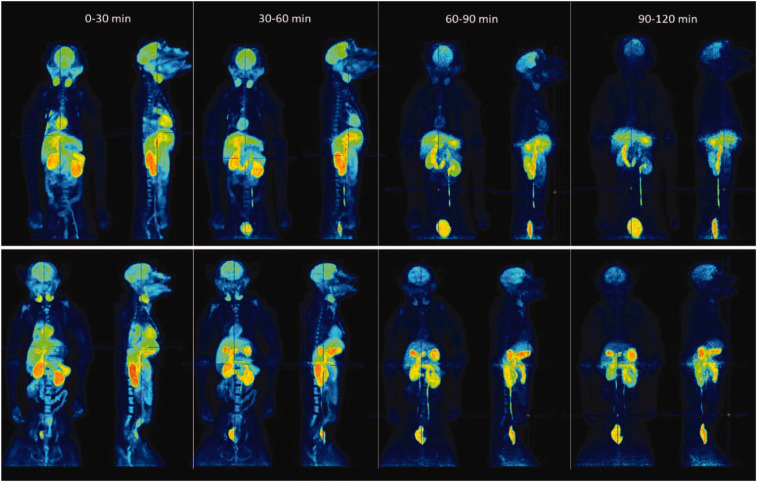
Whole-body PET images of [^11^C]BIO-1819578 in NHP3 and NHP4. Images are decay corrected to time of injection and presented as maximum intensity projection.

The magnitude of the [^11^C]BIO-1819578 uptake was broadly similar between the two NHPs, with the greatest uptake observed in the liver, small intestine, brain, kidneys, heart, and bone, all of which peaked at over 1% of the injected radioactivity. The radioligand was primarily excreted via the bile-gastrointestinal tract, and a low bladder accumulation indicated that the urinary tract was only partly the route of excretion during the scanning window (Figure 5). Residence times for organs are presented in Supplemental Table 2. The largest absorbed dose was in the kidneys (0.014 mGy/MBq) (Supplemental Table 3). The calculated human whole-body effective dose was approximately 0.0033 mSv/MBq (Supplemental Table 3).

## Discussion

The study aimed to quantitatively assess the regional brain distribution of the novel radioligand [^11^C]BIO-1819578 in non-human primates under baseline conditions and after pretreatment with the O-GlcNAcase (OGA) inhibitor Thiamet-G. The PET imaging data showed that the calculated regional *V*_T_ values reflect previous published binding patterns of OGA, with highest binding in hippocampus, striatal regions and the frontal cortex.^13,15^ The 90-minute PET acquisition of [^11^C]BIO-1819578 in NHPs demonstrated predominantly reversible binding across most brain regions (Supplemental Figure 1). However, in select experiments, high-binding areas such as the frontal cortex and hippocampus exhibit slower radioligand clearance (data not shown). The reversibility is a desirable trait for kinetic modelling, distinguishing [^11^C]BIO-1819578 from previously published ^11^C-labeled OGA binding tracers.^13,15^ Although, most time-activity curves showed reversibility, they also exhibited a fast equilibrium between the two tissue compartments and slow wash-out, leading to the collapse of the 2TCM model into 1TCM for almost all brain regions under baseline conditions. The slow kinetics, particularly in the frontal cortex and hippocampus, lead to challenges in the modelling process, which is reflected in the wide distribution of *V*_T_ estimates shown in Figure 3. Analysis of the data reveals that the 1TCM exhibits lower overall test-retest variability compared to other models (Table 2). Additionally, the MA1 model shows promise in reducing variability for the frontal cortex, as evidenced in Table 2. However, the efficacy of this approach in modelling hippocampal binding remains uncertain. The 2TCM performed better based on the AICc score after pretreatment with Thiamet-G, and the most reliable and valid modelling approach will be further evaluated in future human test-retest studies and applied studies focusing on target occupancy with OGA inhibitors.

In this study, *V*_T_ estimated using 2TCM exhibited regional differences with the cerebellum having the lowest values, consistent with saturation studies conducted by Cook et al. in 2023.^15^ No reference region was identified as no region appeared to be devoid of specific binding for OGA. The estimated *V*_T_ decreased in all regions, including the cerebellum, after pretreatment with Thiamet-G, further supporting that no evident reference region could be identified. The high occupancy after pretreatment can be explained by the fact that the blood concentration of Thiamet-G was roughly 1500 times higher than the reported K_i_ value of 20 nM^29^ during the pretreatment scan. The estimated *V*_ND_ was approximately 3 mL·cm^−3^, while the *V*_T_ ranged from 17–41 mL·cm^−3^, indicating that around 84–93% of *V*_T_ represents specific binding.

As reported in by Nag et al.,^18^ the HPLC chromatography analysis of plasma samples did not detect any recirculating lipophilic radiometabolites. Although radiometabolites in the brain cannot be formely excluded, regional brain analysis doesn’t support the presence of radiometabolites confounding quantification. At baseline the PET data could be adequately described using both the one-tissue (1TCM) and two-tissue (2TCM) compartment models, with metabolite-corrected plasma input function. In contrast to [^18^F]-LSN3316612 time-stability estimates in NHPs,^12^ the analysis for [^11^C]BIO-1819578 showed stable estimates of *V*_T_ down to 40–60 minutes, which will be further evaluated in forthcoming human studies. The findings suggest that the formation of blood-brain barrier permeable radiometabolites, which could impact the quantitative analysis, is unlikely but could not be excluded.

Modelling of the parent fraction using a population-based approach allowed for extrapolation of the data up to the full length of the PET examination. However, this approach may introduce some uncertainty in the fitting of the washout phase and the determination of rate constants k_3_ and k_4_, particularly for the three cases where only data up to 60 minutes was available. Nevertheless, the fit as shown in Figure 1 shows good fit also at later time points suggesting that the impact of missing data points is likely low. It is still advised that in later studies sample blood for metabolite analyses every fifteen minutes to ensure as many data points as possible, still using a population-based fit for best results.

The whole-body radiation dosimetry study showed the highest absorbed organ dose was to the kidneys, followed by the gallbladder, heart, and liver. The estimated effective dose (ED) was 0.0033 mSv/MBq of injected radioactivity, which is a relatively low value even for a carbon-11 labelled tracer.^30^ This low ED can likely be attributed to the limited bladder uptake (Supplemental Table 2), which reduces the absorbed dose to nearby radiosensitive organs. It should be noted that the male NHP was castrated and the ED calculation in testis, a region with high OGA expression, did not include any self-dose contribution. Hence, human dosimetry studies are needed for a more accurate testes dose estimation. In future human studies in male subjects the injected activity should be kept low to decrease high ED, as an additional precaution.

## Conclusion

The results in this study supported previous preliminary findings ensuring that [^11^C]BIO-1819578 has promising characteristics as a PET radioligand for imaging OGA. The radioligand demonstrated high uptake in the brain and clear blocking effects when co-administered with the OGA inhibitor Thiamet-G. The baseline data as well as data from blocking studies could reliably be quantified using both gold standard 2TCM as well as the graphical approach MA1. These findings suggest [^11^C]BIO-1819578 shows high potential to be a suitable PET radioligand for quantifying OGA levels in the human brain. The evaluation of effective dose is also an important step prior to first-in-man studies and the levels are such that human administrated is possible. Further testing of this radioligand in human studies is warranted to assess its potential as a tool for studying OGA-related neurological processes and disorders as well as evaluating novel OGA targeting drugs.

## Supplemental Material

sj-pdf-1-jcb-10.1177_0271678X251332487 - Supplemental material for In vivo quantification of [^11^C]BIO-1819578 in non-human primates, a novel radioligand for O-GlcNAcase Supplemental material, sj-pdf-1-jcb-10.1177_0271678X251332487 for In vivo quantification of [^11^C]BIO-1819578 in non-human primates, a novel radioligand for O-GlcNAcase by Martin Bolin, Sangram Nag, Ryosuke Arakawa, Andrea Varrone, Lars Farde, Laurent Martarello, Maciej A Kaliszczak, Christer Halldin and Anton Forsberg Morén in Journal of Cerebral Blood Flow & Metabolism

## References

[1] Holtzman DM Carrillo MC Hendrix JA , et al. Tau: from research to clinical development. Alzheimers Dement 2016; 12: 1033–1039. 27154059 10.1016/j.jalz.2016.03.018

[2] Arendt T Stieler JT Holzer M. Tau and tauopathies. Brain Res Bull 2016; 126: 238–292. 27615390 10.1016/j.brainresbull.2016.08.018

[3] Ossenkoppele R Reimand J Smith R , et al. Tau PET correlates with different Alzheimer’s disease‐related features compared to CSF and plasma p‐tau biomarkers. EMBO Mol Med 2021; 13: e14398. 34254442 10.15252/emmm.202114398 PMC8350902

[4] La Joie R Visani AV Baker SL , et al. Prospective longitudinal atrophy in Alzheimer’s disease correlates with the intensity and topography of baseline tau-PET. Sci Transl Med 2020; 12: eaau5732. 31894103 10.1126/scitranslmed.aau5732 PMC7035952

[5] Basheer N Smolek T Hassan I , et al. Does modulation of tau hyperphosphorylation represent a reasonable therapeutic strategy for Alzheimer’s disease? From preclinical studies to the clinical trials. Mol Psychiatry 2023; 28: 2197–2214. 37264120 10.1038/s41380-023-02113-z PMC10611587

[6] Yuzwa SA Shan X Macauley MS , et al. Increasing O-GlcNAc slows neurodegeneration and stabilizes tau against aggregation. Nat Chem Biol 2012; 8: 393–399. 22366723 10.1038/nchembio.797

[bibr7-0271678X251332487] Hastings NB Wang X Song L , et al. Inhibition of O-GlcNAcase leads to elevation of O-GlcNAc tau and reduction of tauopathy and cerebrospinal fluid tau in rTg4510 mice. Mol Neurodegener 2017; 12: 39. 28521765 10.1186/s13024-017-0181-0 PMC5437664

[8] Yuzwa SA Shan X Jones BA , et al. Pharmacological inhibition of O-GlcNAcase (OGA) prevents cognitive decline and amyloid plaque formation in bigenic tau/APP mutant mice. Mol Neurodegener 2014; 9: 42. 25344697 10.1186/1750-1326-9-42 PMC4232697

[9] Kielbasa W Phipps KM Tseng J , et al. A single ascending dose study in healthy volunteers to assess the safety and PK of LY3372689, an inhibitor of O‐GlcNAcase (OGA) enzyme: human/human trials: anti‐tau. Alzheimer’s & Dementia 2020; 16: e040473.

[bibr10-0271678X251332487] Ryan JM Quattropani A Abd-Elaziz K , et al. O1‐12‐05: phase 1 study in healthy volunteers of the O‐glcnacase inhibitor asn120290 as a novel therapy for progressive supranuclear palsy and related tauopathies. Alzheimer’s & Dementia 2018; 14: P251.

[11] Bartolomé-Nebreda JM Trabanco AA Velter AI , et al. O-GlcNAcase inhibitors as potential therapeutics for the treatment of Alzheimer’s disease and related tauopathies: analysis of the patent literature. Expert Opin Ther Pat 2021; 31: 1117–1154. 34176417 10.1080/13543776.2021.1947242

[12] Paul S Haskali MB Liow JS , et al. Evaluation of a PET radioligand to image *O* -GlcNAcase in brain and periphery of rhesus monkey and knock-out mouse. J Nucl Med 2019; 60: 129–134. 30213846 10.2967/jnumed.118.213231 PMC6354227

[bibr13-0271678X251332487] Lu S Haskali MB Ruley KM , et al. PET ligands [^18^F]LSN3316612 and [^11^ C]LSN3316612 quantify *O*-linked-β-*N*-acetyl-glucosamine hydrolase in the brain. Sci Transl Med 2020; 12: eaau2939. 32404505 10.1126/scitranslmed.aau2939 PMC8494060

[bibr14-0271678X251332487] Wang X Li W Marcus J , et al. MK-8719, a novel and selective *O* -GlcNAcase inhibitor that reduces the formation of pathological tau and ameliorates neurodegeneration in a mouse model of tauopathy. J Pharmacol Exp Ther 2020; 374: 252–263. 32493725 10.1124/jpet.120.266122

[15] Cook BE Nag S Arakawa R , et al. Development of a PET tracer for OGA with improved kinetics in the living brain. J Nucl Med 2023; 64: 1588–1593. 37934021 10.2967/jnumed.122.265225

[16] Varnäs K Varrone A Farde L. Modeling of PET data in CNS drug discovery and development. J Pharmacokinet Pharmacodyn 2013; 40: 267–279. 23660778 10.1007/s10928-013-9320-6

[17] Varrone A Bundgaard C Bang‐Andersen B. PET as a translational tool in drug development for neuroscience compounds. Clin Pharmacol Ther 2022; 111: 774–785. 35201613 10.1002/cpt.2548 PMC9305164

[18] Nag S Bolin M Datta P , et al. Development of a novel [^11^ C]CO-Labeled positron emission tomography radioligand [^11^C]BIO-1819578 for the detection of *O* -GlcNAcase enzyme activity. ACS Chem Neurosci 2023; 14: 2560–2568. 37377046 10.1021/acschemneuro.3c00247 PMC10360070

[19] Ferrat M Dahl K Schou M. One‐pot synthesis of ^11^ C‐labelled primary benzamides via intermediate [^11^C]aroyl dimethylaminopyridinium salts. Chemistry 2021; 27: 8689–8693. 33885193 10.1002/chem.202100544 PMC8251633

[20] Moein MM Nakao R Amini N , et al. Sample preparation techniques for radiometabolite analysis of positron emission tomography radioligands; trends, progress, limitations and future prospects. TrAC Trends in Analytical Chemistry 2019; 110: 1–7.

[21] Giraldo J Vivas NM Vila E , et al. Assessing the (a)symmetry of concentration-effect curves. Pharmacol Ther 2002; 95: 21–45. 12163126 10.1016/s0163-7258(02)00223-1

[22] Gunn RN Gunn SR Cunningham VJ. Positron emission tomography compartmental models. J Cereb Blood Flow Metab 2001; 21: 635–652. 11488533 10.1097/00004647-200106000-00002

[23] Akaike H. Likelihood of a model and information criteria. J Econometr 1981; 16: 3–14.

[24] Ichise M Toyama H Innis RB , et al. Strategies to improve neuroreceptor parameter estimation by linear regression analysis. J Cereb Blood Flow Metab 2002; 22: 1271–1281. 12368666 10.1097/01.WCB.0000038000.34930.4E

[25] Parsey RV Slifstein M Hwang DR , et al. Validation and reproducibility of measurement of 5-HT_1A_ receptor parameters with [*carbonyl* - ^11^C]WAY-100635 in humans: comparison of arterial and reference tissue input functions. J Cereb Blood Flow Metab 2000; 20: 1111–1133. 10908045 10.1097/00004647-200007000-00011

[26] Cselényi Z Olsson H Farde L , et al. Wavelet-aided parametric mapping of cerebral dopamine D2 receptors using the high affinity PET radioligand [11C]FLB 457. NeuroImage 2002; 17: 47–60. 12482067 10.1006/nimg.2002.1152

[27] Cunningham VJ Rabiner EA Slifstein M , et al. Measuring drug occupancy in the absence of a reference region: the lassen plot re-visited. J Cereb Blood Flow Metab 2010; 30: 46–50. 19738632 10.1038/jcbfm.2009.190 PMC2949110

[28] Stabin MG Sparks RB Crowe E. OLINDA/EXM: the second-generation personal computer software for internal dose assessment in nuclear medicine. J Nucl Med 2005; 46: 1023–1027. 15937315

[29] Yuzwa SA Macauley MS Heinonen JE , et al. A potent mechanism-inspired O-GlcNAcase inhibitor that blocks phosphorylation of tau in vivo. Nat Chem Biol 2008; 4: 483–490. 18587388 10.1038/nchembio.96

[30] Zanotti-Fregonara P Innis RB. Suggested pathway to assess radiation safety of 11C-labeled PET tracers for first-in-human studies. Eur J Nucl Med Mol Imaging 2012; 39: 544–547. 22160195 10.1007/s00259-011-2005-8 PMC3791506

