# Supplementary material for: In vivo quantification of [11C]BIO-1819578 in non-human primates, a novel radioligand for O-GlcNAcase
Source: J Cereb Blood Flow Metab. 2025 Apr 12;45(8):1606–16. doi: 10.1177/0271678X251332487 (PMC11994644; doi:10.1177/0271678X251332487)
Supplement: sj-pdf-1-jcb-10.1177_0271678X251332487 - Supplemental material for In vivo quantification of [11C]BIO-1819578 in non-human primates, a novel radioligand for O-GlcNAcase [file sj-pdf-1-jcb-10.1177_0271678X251332487.pdf]

Supplemental

All together five baseline PET-measurements were performed in the three NHPs. The time-activity curves for the whole brain peaked at approximately 4.4 SUV on average around 15 minutes post radioligand injection and then decreased to around 3.3 SUV at 90 minutes under baseline conditions. Average regional uptake curves at baseline (n=5) are presented in Supplemental Figure 1; Regional brain uptake generally peaked at 5-15 minutes post-injection.

The time curve for total radioactivity in arterial plasma was generated using the continuous whole blood and discrete blood and plasma samples. Blood to plasma ratio was best estimated using an exponential fitting method. Extrapolation of the parent fraction estimation, using a population-based approach, was carried out, as low signal to noise was observed chromatogram analysis. On average, the model estimated that 26% of [^{11}C]BIO-1819578 remained unmetabolized in NHP plasma at 90 minutes post-injection (Supplemental Figure 2). The time curve for parent fraction and the blood to plasma ratio was used to correct the total radioactivity in arterial blood to obtain the metabolite corrected arterial input function used in the compartment analysis.

In the analysis of baseline scans, the 2TCM demonstrated that V_T values across different brain regions converged to within 10% of their final values after 40-60 minutes of image acquisition (Supplemental Figure 3).

For the pretreatment data, all regions were preferably modelled using the 2TCM according to the AICc score, and the R^2 between the two compartmental approaches was 0.93. After pretreatment with Thiamet-G, an occupancy of 97.6 % (calculated using the 2TCM) was observed, with an estimated V_{ND} of 2.9 mL·cm^{-3} (Supplemental Figure 4).

At baseline, both the 1-tissue compartment model (1TCM) and the 2-tissue compartment model (2TCM) were able to describe the time-activity curves in most brain regions (Figure 1), and the Akaike information criterion (AICc) could not separate out a preferable model across regions and subjects.

The magnitude of the [^{11}C]BIO-1819578 uptake was broadly similar between the two NHPs, with the greatest uptake observed in the liver, small intestine, brain, kidneys, heart, and bone, all of which peaked at over 1% of the injected radioactivity. The radioligand was primarily excreted via the bile-gastrointestinal tract, and a low bladder accumulation indicated that the urinary tract was only partly the route of excretion during the scanning window (Figure 5). The numbers of disintegrations in the source organs are presented in Supplemental Table 2. The largest absorbed dose was in the kidneys (0.014 mGy/MBq) (Supplemental Table 3). The calculated human whole-body effective dose was approximately 0.0033 mSv/MBq (Supplemental Table 3).

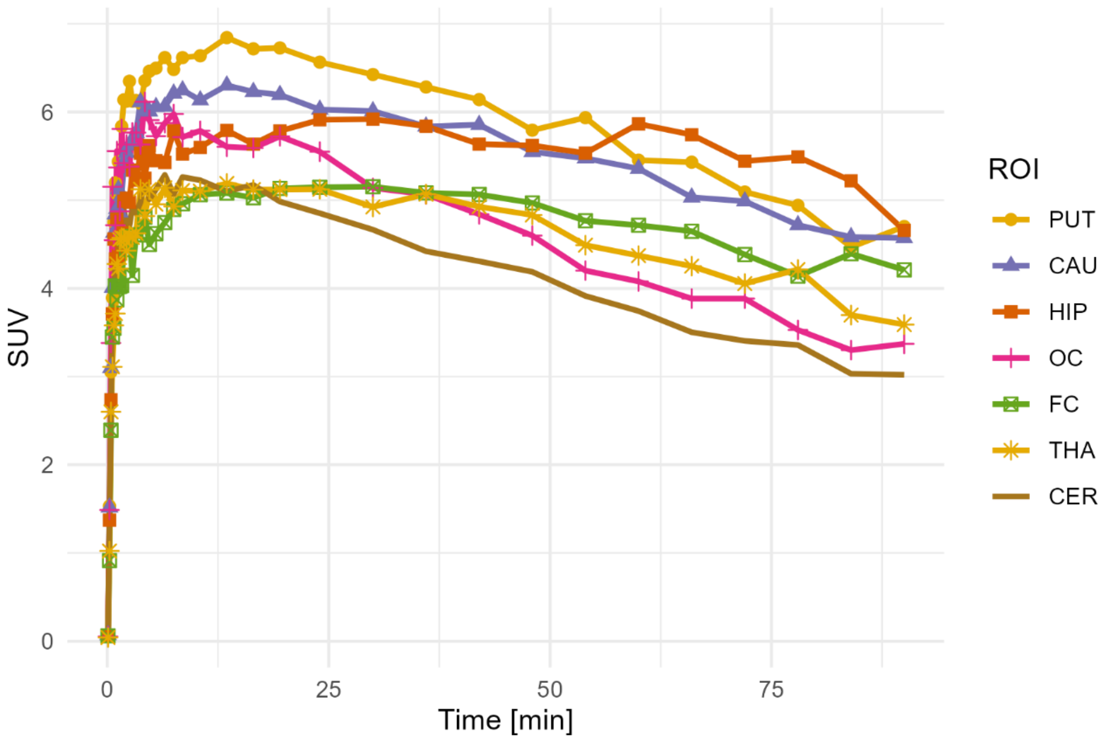

Supplemental Figure 1: Regional brain time–activity curves of [^{11}C]BIO-1819578 at baseline. Presented are average SUV TACs (n=5). PUT: putamen; CAU: caudate; HIP: hippocampus; OC: occipital cortex; FC: frontal cortex; THA: thalamus; CER: cerebellum

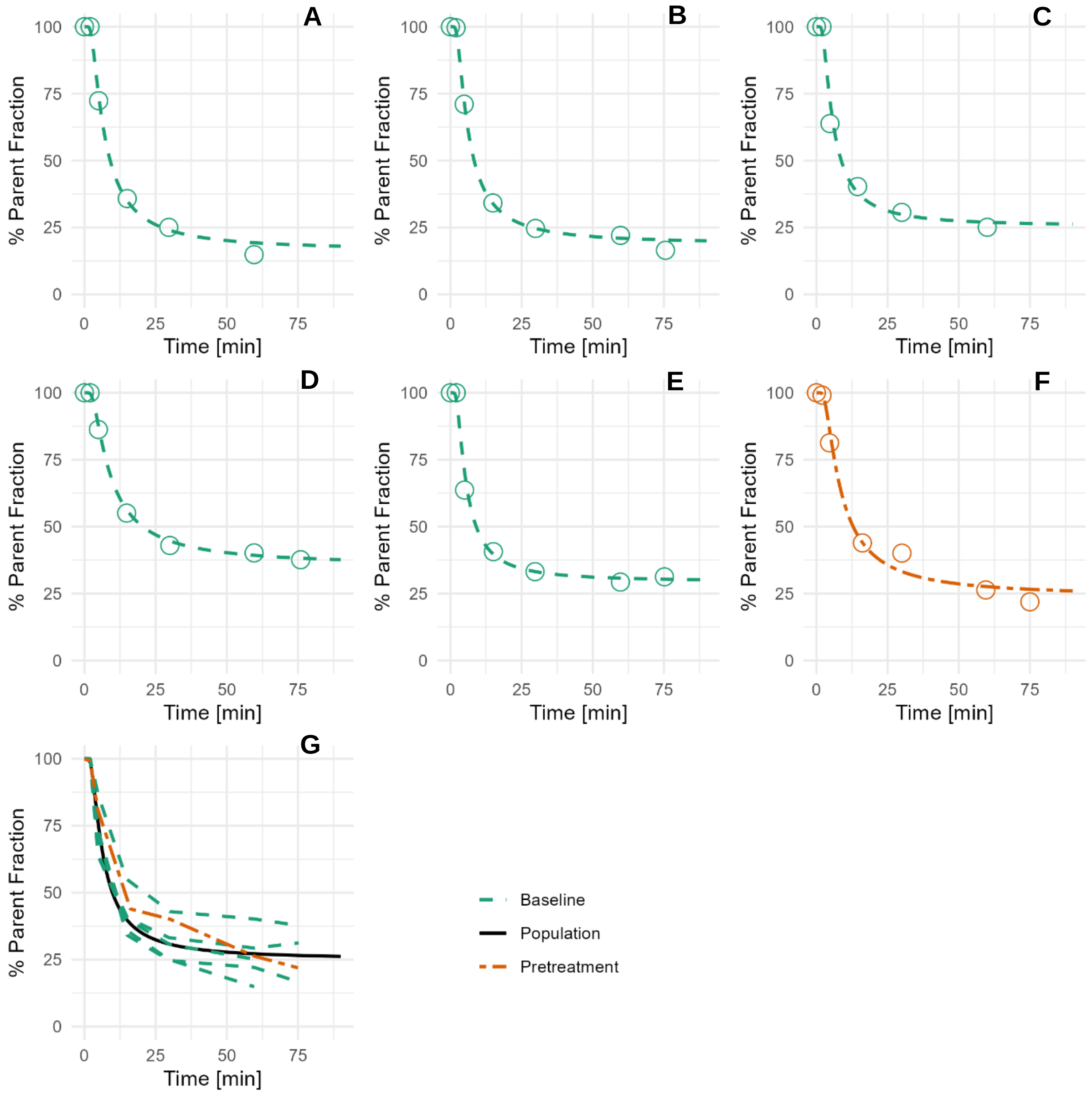

Supplemental Figure 2: Individual parent Fraction fits and population model using Hill function. Each panel displays one PET experiment, except for the last panel (G) in which all calculated data and the population average are presented. In other panels (A-F), circles indicate calculated parent fractions, and the lines shows the estimated values used in the modelling.

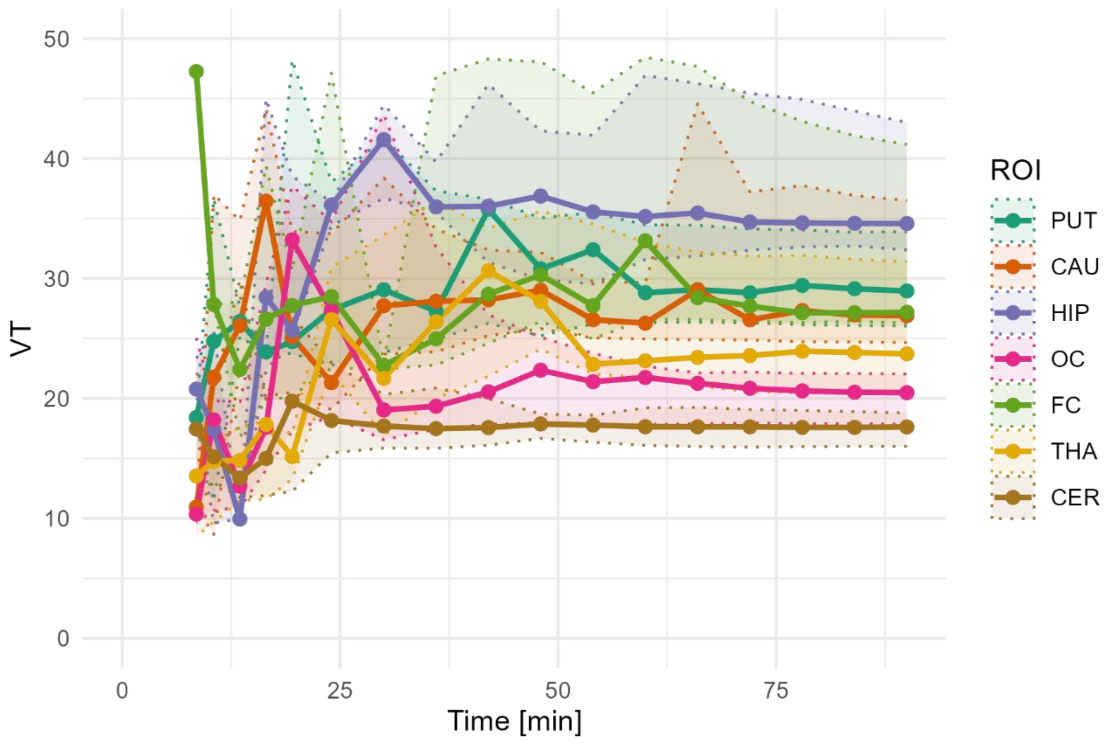

Supplemental Figure 3: Time-stability graphs for the calculated regional V_T values. V_T remains stable down to 40-60 minutes. CAU: caudate; CER: cerebellum; FC: frontal cortex; HIP: hippocampus; OC: occipital cortex; PUT: putamen; THA: thalamus.

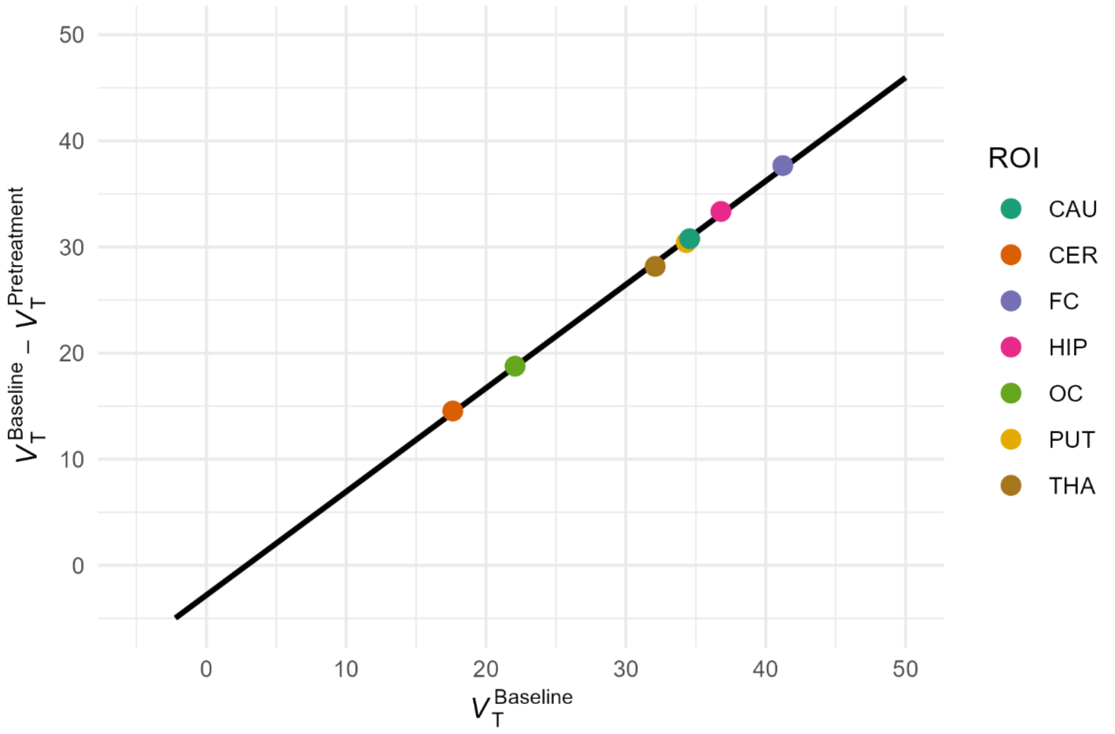

Supplemental Figure 4: Revisited Lassen plot from baseline and pretreatment PET of NHP3. Solid line is the calculated linear fit. CAU: caudate; CER: cerebellum; FC: frontal cortex; HIP: hippocampus; OC: occipital cortex; PUT: putamen; THA: thalamus.

Supplemental Table 1: Δ AICc values for different PET experiments and regions. A positive value indicates that 2TCM is the preferred model.

	PUT	CAU	FC	OC	HIP	CER	THA
**NHP1, Test**	49.4	50.7	31.2	47.9	22.1	45.8	31.1
**NHP1, Retest**	26.0	16.3	24.1	35.5	27.6	44.5	25.7
**NHP2, Test**	-5.1	-5.0	-5.0	-4.0	0.9	-4.8	-3.4
**NHP2, Retest**	4.7	-4.2	-5.0	-4.8	-3.6	-5.1	-1.5
**NHP3, Baseline**	-5.1	11.8	-5.1	0.9	-5.1	-5.1	-5.1
**NHP3, Pretreatment**	59.6	81.6	51.9	69.9	38.0	73.9	14.3

Supplemental Table 2: Residence times for organs in each animal and their mean for [^{11}C]BIO-1819578 (MBq.h/MBq)

**Organ**	**Animal 1**	**Animal 2**	**Mean**
**Heart**	0.0077	0.0083	0.0080
**Lungs**	0.0055	0.0032	0.0044
**Brain**	0.0175	0.0184	0.0179
**Liver**	0.0379	0.0345	0.0362
**Thyroid**	0.0001	0.0001	0.0001
**Bone**	0.0051	0.0056	0.0054
**Salivary glands**	0.0019	0.0016	0.0017
**Kidneys**	0.0144	0.0144	0.0144
**Urinary bladder**	0.0027	0.0040	0.0033
**Spleen**	0.0018	0.0021	0.0020
**Small intestine**	0.0352	0.0261	0.0306
**Gallbladder**	N/A	0.0034	0.0034
**Organ total**	0.1297	0.1217	0.1257
**Remaining activity**	0.3592	0.3672	0.3632

Supplemental Table 3: Organ and effective doses to the adult hermaphrodite phantom for [^{11}C]BIO-1819578

**Target Organ**	**Organ Dose (mGy/MBq)**			**Effective Dose (mSv/MBq)**
	Beta	Gamma	Total	
**Adrenals**	1.09E-03	2.11E-03	3.21E-03	8.02E-06
**Brain**	2.81E-03	1.69E-03	4.51E-03	1.13E-05
**Breasts**	1.09E-03	9.74E-04	2.07E-03	1.03E-04
**Gallbladder Wall**	7.80E-03	3.63E-03	1.14E-02	0.00E+00
**LLI Wall**	1.09E-03	2.00E-03	3.09E-03	3.71E-04
**Small Intestine**	9.41E-03	2.72E-03	1.19E-02	2.97E-05
**Stomach Wall**	1.09E-03	1.84E-03	2.93E-03	3.52E-04
**ULI Wall**	1.09E-03	2.79E-03	3.89E-03	9.72E-06
**Heart Wall**	5.76E-03	2.36E-03	8.12E-03	0.00E+00
**Kidneys**	1.07E-02	3.64E-03	1.43E-02	3.58E-04
**Liver**	4.23E-03	2.96E-03	7.18E-03	3.59E-04
**Lungs**	1.02E-03	1.38E-03	2.40E-03	2.89E-04
**Muscle**	1.09E-03	1.34E-03	2.44E-03	6.09E-06
**Ovaries**	1.09E-03	2.31E-03	3.41E-03	6.81E-04
**Pancreas**	1.09E-03	2.22E-03	3.31E-03	8.28E-06

**Red Marrow**	1.08E-03	1.60E-03	2.68E-03	3.21E-04
**Osteogenic Cells**	2.33E-03	1.56E-03	3.89E-03	3.89E-05
**Skin**	1.09E-03	8.56E-04	1.95E-03	1.95E-05
**Spleen**	2.37E-03	1.99E-03	4.37E-03	1.09E-05
**Testes**	1.09E-03	1.18E-03	2.27E-03	0.00E+00
**Thymus**	1.09E-03	1.40E-03	2.49E-03	6.24E-06
**Thyroid**	1.06E-03	1.27E-03	2.33E-03	1.17E-04
**Urinary Bladder Wall**	2.36E-03	1.85E-03	4.21E-03	2.10E-04
**Uterus**	1.09E-03	2.27E-03	3.36E-03	8.41E-06
**Total Body**	1.44E-03	1.42E-03	2.86E-03	0.00E+00
**Total Effective Dose (mSv/MBq)**				**3.32E-03**
